# Incidence of HER2-targeted antibody-drug conjugates-related cardiac events: a meta-analysis

**DOI:** 10.7150/jca.90090

**Published:** 2024-01-01

**Authors:** Fen Liu, Huamin Li, Guisen Yin, Yong Pan

**Affiliations:** 1 Department of Pharmacy, Hunan Cancer Hospital, the Affiliated Cancer Hospital of Xiangya School of Medicine, Central South University, Changsha 410011, Hunan, China.; 2 Department of Pharmacy, the Second Xiangya Hospital, Central South University, Changsha 410011, Hunan, China.; 3 Department of Pharmacy, Yantai Hospital of Traditional Chinese Medicine, Yantai 264000, Shandong, China.

**Keywords:** human epidermal growth factor receptor 2, antibody-drug conjugate, cardiac events, trastuzumab emtansine, trastuzumab deruxtecan

## Abstract

**Background**: Human epidermal growth factor receptor 2 (HER2)-targeted antibody-drug conjugate (ADC) has emerged as a hotspot for research and brought breakthroughs in the treatment of breast cancer and other solid tumors. While the occurrence of cardiac events (CEs) has yet not been systematically reported.

**Methods**: The prospective clinical trials of marketed HER2-targeted ADCs were systematically searched in PubMed, Embase, Cochrane Library, and ClinicalTrials.gov from inception to May 2023. Two investigators independently extracted data with priority given to ClinicalTrials.gov, followed by peer-reviewed articles. Stata 15.0 software was used to perform the meta-analysis. The effect statistics were estimated as pooled incidence with 95% confidence intervals (CI). The primary objectives were to assess the incidence of all-grade and ≥3 /serious grades CEs related to HER2-targeted ADC. Our study strictly adhered to the Preferred Reporting Items for Systematic Reviews and Meta-Analyses (PRISMA) guidelines and has been registered on PROSPERO (NO. CRD42023440448).

**Results**: After conducting a comprehensive literature search, initially 7000 relevant studies were identified, and eventually a total of 47 trials involving 10594 patients were included for analysis. The pooled incidence of all-grade and ≥3/serious grades CEs respectively were 4.7% [95% CI, 3.7-5.8%] and 0.6% (95% CI, 0.5-0.8%). The pooled incidence of CEs leading to dosage discontinuation was 0.8% (95% CI, 0.4-1.3%). Subgroup analysis revealed a significantly higher incidence of all-grade CEs in T-DXd treatment compared to T-DM1 treatment (7.7% versus 3.6%; *p*=0.017), as well as in phase I/II trials compared to phase III trials (6.9% versus 3.2%; *p*=0.002) and combination therapy compared to monotherapy (7.6% versus 3.9%; *p*=0.013). The electrocardiogram QT corrected interval prolonged was identified as the CE with the highest pooled incidence, occurring at a rate of 5.9% (95% CI, 3.3-8.5%).

**Conclusions**: The incidence of CEs associated with HER2-targeted ADC is relatively low. However, it is crucial to enhance surveillance measures, particularly for T-DXd treatment and combination therapy.

## Introduction

Antibody-drug conjugate (ADC) is a new class of antineoplastic drugs with a special structure different from conventional chemotherapy drugs, containing monoclonal antibodies, cytotoxic drugs and chemical linkers. It has emerged as a hotspot for research and development of antineoplastic drugs. Fifteen ADCs have been approved for the market so far worldwide^1^, of which three were human epidermal growth factor receptor 2 (HER2)-targeted ADC. The first HER2-targeted ADC, trastuzumab emtansine (T-DM1), was initially approved in 2013 for HER2-positive metastatic breast cancer^2^. Its indication was later expanded in 2019 to the adjuvant therapy for early-stage HER2-positive breast cancer^3^. The second HER2-targeted ADC, trastuzumab deruxtecan (T-DXd, formerly DS-8201a), received FDA approval in 2019 as a late-line treatment for unresectable or metastatic HER2-positive breast cancer^4^. Subsequently, its usage has been extended to HER2-low (IHC 1+ or IHC 2+/ISH-) breast cancer^5^, HER2-positive gastric or gastroesophageal (GEJ) adenocarcinoma^6^, and unresectable or metastatic non-small cell lung cancer with HER2 mutation^7^. The third HER2-targeted ADC, disitamab vedotin (RC48), received approval in 2021 for locally advanced or metastatic gastric or gastroesophageal junction cancer and urothelial carcinoma from the National Medical Products Administration (NMPA) of China^8^. HER2-targeted ADCs are changing the destiny of HER2-expressing solid tumors^9^.

The imbalance of cell signaling in the HER family, including HER1 (also known as EGFR), HER2, HER3 and HER4, has been associated with the occurrence and development of multiple tumor types^10^. The advent of trastuzumab, the first humanized monoclonal antibody targeting HER2, has revolutionized the treatment landscape and brought a significant breakthrough for HER2-positive breast cancer. Trastuzumab has firmly established itself as a cornerstone of adjuvant, neoadjuvant therapy and the systemic treatment for HER2-positive breast cancer for over two decades. With the progressive advancement of HER2-targeted therapeutics, the cardiotoxicity associated with these agents has garnered significant attention. A meta-analysis^11^, which included ten randomized controlled trials (RCTs) with a total of 11,882 patients, revealed that trastuzumab significantly increased the risk of Left Ventricular Ejection Fractions (LVEF) decrease (RR = 2.13, 95% CI, 1.31-3.49; *p* = 0.003), as well as the risk of congestive heart failure (CHF) (RR = 4.19, 95% CI 2.73-6.42; *p* <0.001). Cardiac events (CEs) associated with other HER2-targeted agents have also been sporadically documented^12^. The EMILIA research indicated that the incidence of ≥3 grades cardiac dysfunction associated with T-DM1 was less than 1%, which was significantly lower than trastuzumab^13^, and T-DXd exhibited similar findings^14^. The low incidence of such events makes small-scale studies inadequate for fully capturing the characteristics of HER2-targeted ADC-induced CEs. The impact of factors, including different drugs and drug concentrations, monotherapy or combination therapy, tumor types and stages, on the incidence of CEs associated with HER2-targeted ADC remains uncertain. Therefore, we thoroughly conducted a comprehensive systematic review and literature analysis of all prospective clinical studies on HER2-targeted ADCs to investigate the incidence of CEs at all grades and ≥3/serious grades, and identify the factors influencing these events.

## Methods

### Search strategy and selection criteria

The study protocol for our meta-analysis had been registered on PROSPERO (NO. CRD42023440448) and was conducted following the Preferred Reporting Items for Systematic Reviews and Meta-Analyses (PRISMA) guidelines^15^. The prospective clinical trials on HER2-targeted ADCs were retrieved from PubMed, Embase, Cochrane Library and ClinicalTrials.gov, with a search period ranging from the database's inception to May 2023, and only English-language publications were included. Detailed search strategies were provided in Table S1. The clinical trials meeting the following criteria were included: (1) prospective clinical trials; (2) participants who received monotherapy or combination therapy of the approved HER2-targeted ADC; (3) available count data regarding treatment-related cardiotoxicity. Exclusion criteria: (1) studies with a sample size of less than ten participants; (2) trial in progress or no available date of CEs; (3) duplicate studies.

### Data extraction

The trials were independently searched and screened by two researchers (Fen Liu and Huamin Li) according to inclusion and exclusion criteria. Any disagreements were resolved by a third researcher, Guisen Yin. The existence of reporting discrepancies between the results on ClinicalTrials.gov and those in peer-reviewed publications is widely acknowledged^16^. Given that ClinicalTrals.gov provides comprehensive reporting of all adverse events (AEs) and regularly updates them even after publication. Moreover, limited data are available in peer-reviewed publications, so we prioritized extracting data from ClinicalTrials.gov followed by articles. If multiple identical articles exist for the same sample, only the one with the most comprehensive documentation for CEs was chosen. Additionally, we also considered contacting the authors for data if necessary. The study was excluded if data could not be obtained. The following information was extracted: first author, publication year, study name, study types, study phase, NCT numbers, HER2-targeted ADCs used and their dosage, cancer type and cancer status, number of patients in the safety analysis, and number of all-grade and ≥3/serious grades CEs related to HER2-targeted ADC. The high-grade CEs reported in publication and serious CEs reported in ClinicalTrials.gov were pooled for a meta-analysis.

### Study objective

The primary objectives were to assess the incidence of all-grade and ≥3/serious grades CEs related to HER2-targeted ADC and the occurrence of CEs leading to dosage discontinuation. The secondary objectives aimed to analyze the incidence of all-grade and ≥3 or serious grades CEs based on different drugs and drug concentrations, monotherapy or combination therapy, study phase, tumor types and tumor status.

### Statistical analysis

Stata 15.0 software was used to perform the meta-analysis. The effect statistics were estimated as pooled incidence with 95% confidence intervals (CI). A combination of Chi-square test (α=0.10) and I^2^ value was employed to assess heterogeneity among the trials. If I^2^>50% or *p*<0.10, it indicated substantial heterogeneity across studies, necessitating the adoption of a random effects model. Subgroup analysis was conducted to compare the differences between subgroups, *p*<0.05 meant statistical difference. The methodological quality of literature was independently assessed by two researchers (Fen Liu and Huamin Li) using the Cochrane risk-of-bias tool (ROB) for randomized controlled trials (RCTs) and the Methodological index for non-randomized studies (MINORS) for non-RCTs^17^. Any inconsistencies were resolved through discussion with a third researcher (Guisen Yin). The stability of the pooled incidence estimate was evaluated through a sensitivity analysis, in which each literature was systematically omitted one by one. Additionally, publication bias in the included studies was assessed using a funnel plot and Egger's test (α=0.05).

## Results

### Study selection and characteristics

**Figure 1:**
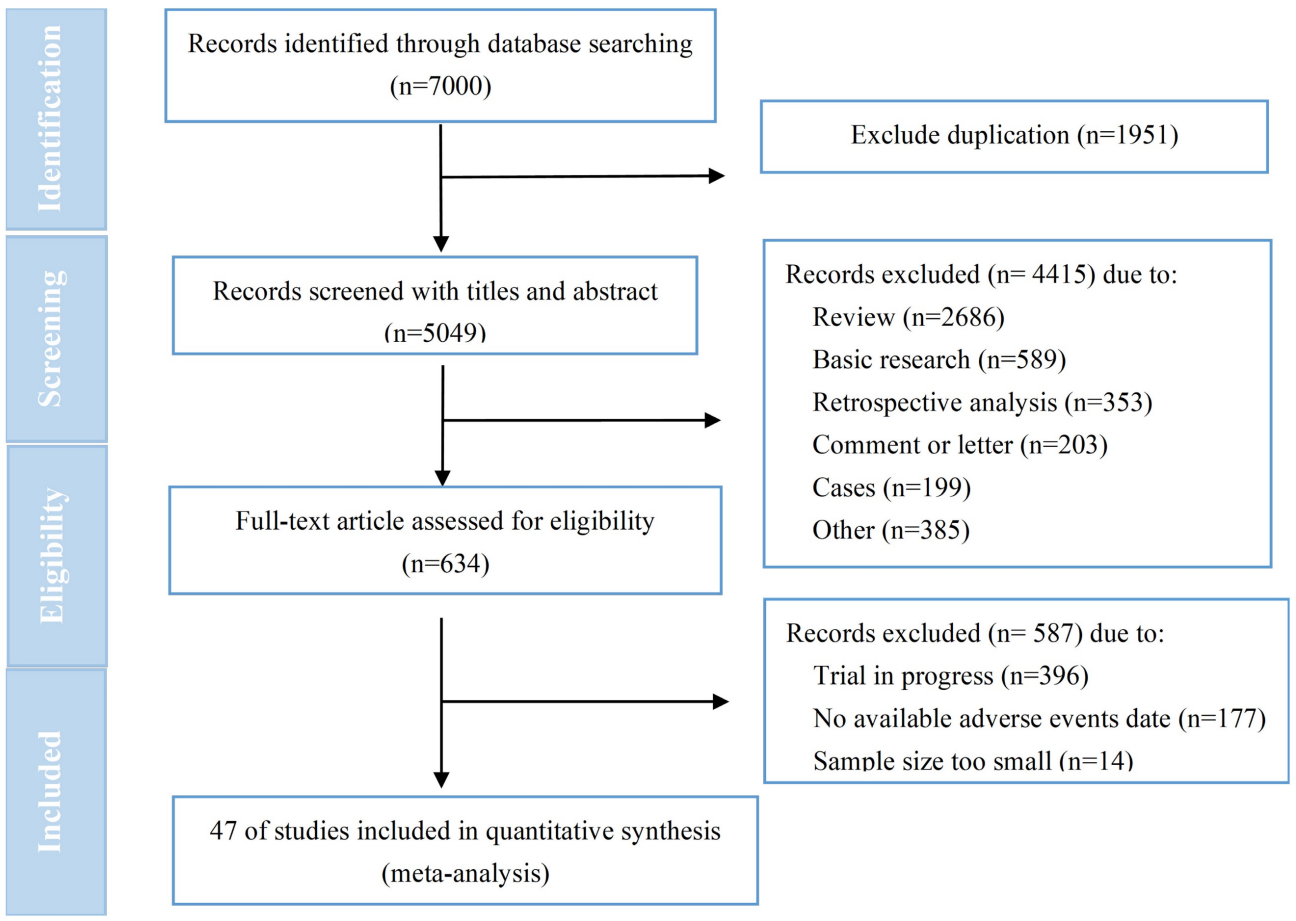
Flow diagram of included studies.

7000 relevant studies were identified after conducting a comprehensive literature search. Following a stepwise screening procedure outlined in Figure 1, a total of 47 trials^7^, ^13^, ^14^, ^18^-^65^ were selected for analysis. No one was for RC48 due to a lack of available data. The quality assessment showed that all RCTs (n=16) were classified as high risk, primarily due to the absence of blinding among participants, personnels and assessors (Figure S1). The MINORS scores for non-RCTs (n=31) ranged from 8 to 21, with a mean score of 11.5 (Table S2). Failure to implement blinded evaluation was frequently identified as a common deficiency for non-RCTs. According to established principles of data extraction, we extracted data from ClinicalTrials.gov for 27 trials and published articles or abstracts for 20 trials. The general characteristics of included trials were presented in Table 1. T-DM1 was assessed in 37 trials (n=8664), T-DXd was assessed in 11 trials (n=1930), T-DXd and T-DM1 evaluated within the same study^19^. The 37 trials related to T-DM1 consisted of 19 monotherapy trials, 14 combination therapy trials, and 4 trials that involved both monotherapy and combination therapy. It was predominantly administered in advanced/metastatic tumors (30/37). Breast cancer emerged as the most prevalent tumor type for T-DM1 application (32 /37), followed by lung cancer (3 /37), gastric cancer (2 /37), and other solid tumors (1/37). Among these, 13 were randomized controlled trials (RCT) and 24 were non-RCT. The 11 trials on T-DXd consisted of 4 RCTs and 7 non-RCTs. These trials were all conducted in patients with advanced/metastatic disease (11/11), primarily focusing on monotherapy (10/11). As well, breast cancer emerged as the most prevalent tumor type for T-DXd application (8/11), followed by gastric cancer (3 /11), other solid tumors (2/11), and lung cancer (1 /11).

### The incidence of HER2-targeted ADC-related CEs

**Figure 2:**
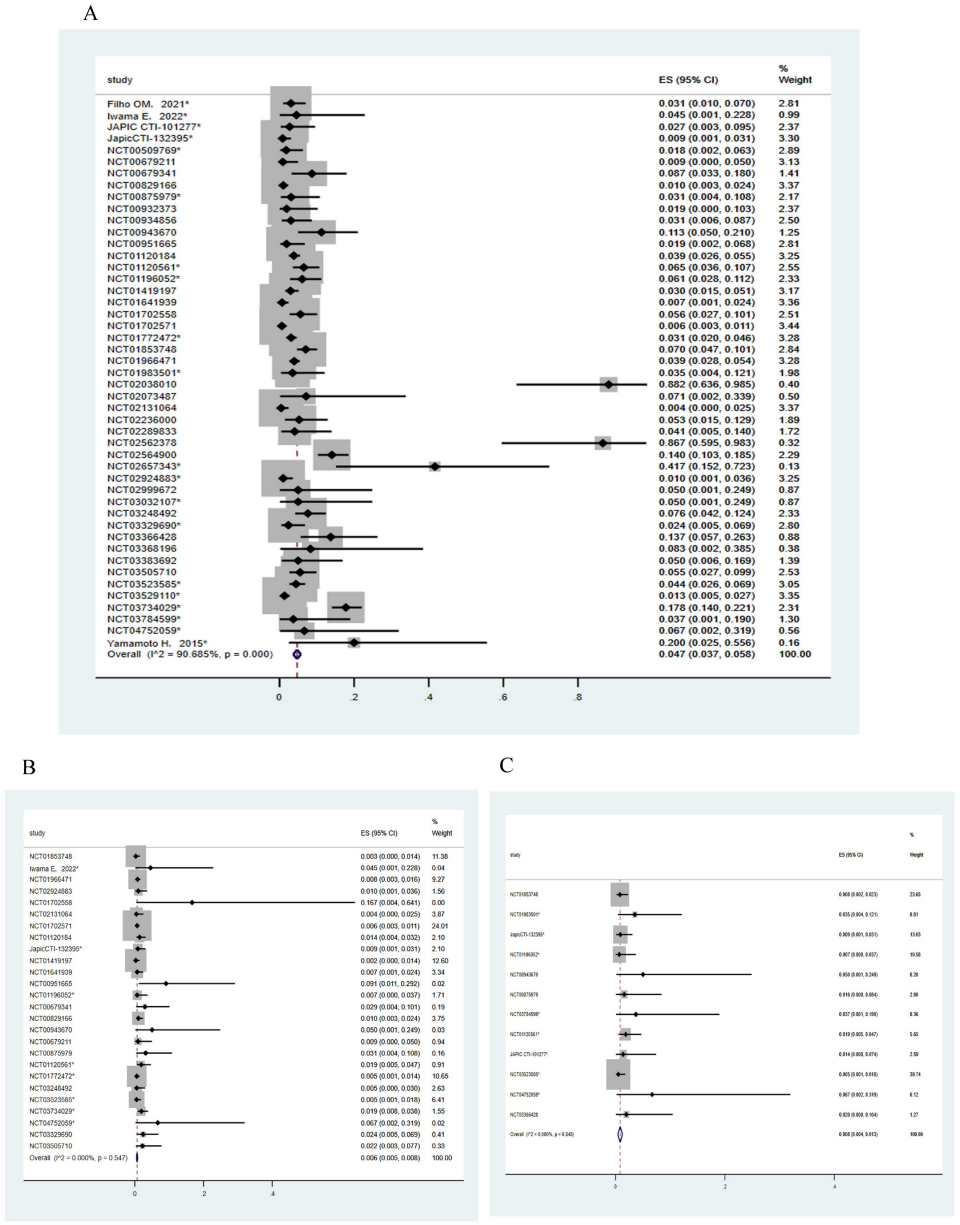
The incidence of ADC-related cardiac events. (A) The incidence of all-grade ADC-related cardiac events in 47 trials. (B) The incidence of ≥3/serious grades ADC-related cardiac events in 26 trials. (C) The incidence of ADC-related cardiac events resulting in the dosage discontinuation in 12 trials

A total of 47 trials involving 10594 patients and 26 trials involving 8112 patients were included for analyzing the incidence of all-grade and ≥3/serious grades HER2-targeted ADC-related CEs, respectively. The incidence of CEs leading to dosage discontinuation was analyzed in 12 trials involving 1691 patients. The random effects model was employed for meta-analysis due to the substantial heterogeneity. As shown in Figure 2, the incidence of CEs at all-grade and ≥3/serious grades respectively were 4.7% (95% CI, 3.7-5.8%) and 0.6% (95% CI, 0.5-0.8%). The incidence of CEs leading to dosage discontinuation was 0.8% (95% CI, 0.4-1.3%).

### The incidence of all-grade CEs across different subgroups

#### The incidence of all-grade CEs across different drugs

The incidence of all-grade CEs associated with T-DM1 and T-DXd were respectively assessed in 37 trials and 11 trials. The incidence of all-grade CEs associated with T-DXd was 7.7% (95% CI, 4.5-10.9%), which was significantly higher than that of T-DM1[3.6% (95% CI, 2.6-4.6%)](*p*=0. 017)(Table 2).

#### The incidence of all-grade CEs between phase I/II trials and phase III trials

A total of 35 phase I/II trials and 10 phase III trials were included for analysis. Two trials were excluded, one with the unknown study phase (NCT01120561) and another with phase II/III (NCT01641939). The incidence of all-grade CEs in phase III trials was 3.2% (95% CI, 1.9-4.5%), which was significantly lower than that of phase I/II trials[6.9% (95% CI, 4.9-8.9%)] (*p*=0.002) (Table 2). Further, a subgroup analysis of various drugs was performed. Similarly, the incidence of all-grade CEs associated with T-DM1 in phase III trials was 1.9% (95% CI, 1.0-2.8%), which was significantly lower than that of phase I/II trials[6.6% (95% CI, 4.4-8.9%)] (*p*<0.001) (Figure S2A). However, the incidence of all-grade CEs associated with T-DXd between phase I/II and III trials were similar, with rates of 7.6 (95% CI, 3.9-11.2%) and 8.1% (95 %CI, 1.2-14.9%) (*p*=0.906) (Figure S2B ).

#### The incidence of all-grade CEs between monotherapy and combination therapy

47 trials were included in the analysis, with 4 of them (NCT02924883, NCT01702558, NCT01120184, NCT00943670) examining both monotherapy and combination therapy. The data from these four trials were further divided, resulting in 32 studies assessing monotherapy and 19 studies evaluating combination therapy. As shown in Table 2, the incidence of all-grade CEs in combination therapy was 7.6% (95% CI, 4.9-10.4%), which was significantly higher than that of monotherapy at the rate of 3.9% (95% CI, 3.0-4.9%) (*p*=0.013). Data on monotherapy were further analyzed according to drug subgroup. As presented in Figure S3A, the incidence of all-grade CE in T-DXd monotherapy treatment was 8.8% (95%CI, 4.9-12.7%), which was significantly higher than that of T-DM1 monotherapy treatment at the rate of 2.3% (95% CI, 1.5-3.0%) (*p*=0.001). As presented in Figure S4A, the incidence of all-grade CEs in T-DM1 combination therapy was 7.8% (95 %CI, 5.0-10.7%), which was significantly higher than that of monotherapy at a rate of 2.3% (95 %CI, 1.5-3.0%) (*p*<0.001).

#### The incidence of all-grade CEs in different solid tumor

Further, the CEs of various tumor types were analyzed. The two trials (NCT01702558, NCT02564900) were analyzed separately for breast and gastric cancer. The trial (NCT03368196) was excluded from this analysis due to the inability to separate data from breast and gastric cancer. The final analysis results were shown in Table 2. The incidence of all-grade CEs was 5.5% (95 %CI, 3.7-7.6%) for breast cancer, 4.5% (95% CI, 2.1-7.5%) for lung cancer, 3.0% (95% CI, 0.0-9.1%) for gastric cancer, and 4.8% (95% CI, 0.3-12.5%) for other solid tumors.

Breast cancer emerged as the most prevalent tumor type in our meta-analysis. Subsequently, we conducted a subgroup analysis based on the tumor status of breast cancer. As shown in Table 2, the incidence of all-grade CEs was 3.7% (95% CI, 1.8-5.6%) for early-stage breast cancer and 5.8% (95% CI, 4.3-7.3%) for advanced/metastatic breast cancer, with no statistical differences (*p*=0.093).

#### The incidence of all-grade CEs between different dosages for T-DM1 and T-DXd

The incidence of CEs of various dose subgroups for T-DM1 and T-DXd was analyzed. As shown in Table 2, the incidence of all-grade CEs of T-DM1 was 4.3% (95% CI, 2.7-6.2 %) for 3.6mg/kg treatment and 3.8% (95 %CI, 0.0-19.1%) for less than 3.6mg/kg treatment, with no statistical differences (*p*=0.933). Likewise, the incidence of all-grade CEs of T-DXd was 7.2% (95% CI, 2.8-13.0%) for 5.4mg/kg treatment and 7.1% (95% CI, 1.4-15.9%) for 6.4 mg/kg treatment, with no statistical differences (*p*=0.933).

### The incidence of ≥3/serious grades CEs across different subgroups

Table 3 showed no statistically significant difference in the incidence of ≥3/serious grades CEs between T-DXd and T-DM1, phase I/II and III trials, monotherapy and combination therapy, different tumor types and tumor status. However, a trend towards a higher incidence of ≥3/serious grades CEs was observed in T-DXd compared to T-DM1 (1.0% versus 0.6%; *p*=0.307), as well as in advanced/metastatic breast cancer compared to early-stage breast cancer (0.7% versus 0.5%; *p*=0.379) (Table 3).

### Types of CEs associated with HER2-targeted ADC

The CEs documented in the 47 literatures underwent analysis. As presented in Table 4, a total of 440 cases and 73 cases of CEs at all grades and ≥3/serious grades CEs were respectively documented. The incidence of special CEs reported in more than three trials was pooled and analyzed, while only descriptive analysis was performed for those reported in three or fewer trials. The analysis revealed that electrocardiogram QT corrected interval prolonged was identified as the CE with the highest pooled incidence, at a rate of 5.9% (95% CI, 3.3-8.5%).

Additionally, the ejection fraction decreased emerged as the highest proportion of all grades CEs, accounting for 39.3% (173/440), and cardiac failure (congestive) emerged as the most proportion of ≥3/serious grades CEs with a ratio of 19.2% (14/73).

### Sensitivity analysis and publication bias

After excluding single literature, the incidence of all-grade CEs remained consistent with the incidence prior to literature exclusion, indicating the stability and reliability of the meta-analysis results. Funnel plots were utilized to visually assess the publication bias in the literature included within this study. As depicted in Figure S5, the meta-analyses examining the incidence of all grades and ≥3/serious grades CEs exhibited significant publication bias, which were further confirmed by the Egger's test (*p*<0.001).

## Discussion

To our knowledge, this meta-analysis comprehensively evaluated the incidence of CEs associated with commercially available HER2-targeted ADCs first. Our research indicated that the pooled incidence of all grades and ≥3/serious grades CEs associated with HER2-targeted ADC were 4.7% and 0.6%. The pooled incidence of CEs leading to dosage discontinuation was 0.8%. A significantly higher incidence of all-grade CEs was revealed in T-DXd treatment compared to T-DM1 treatment, as well as in phase I/II trials compared to phase III trials and combination therapy compared to monotherapy. No statistical difference was observed in the incidence of CEs across different solid tumors, various dosages of T-DM1 and T-DXd, and tumor status at all grades. The occurrence of ≥3/serious grades CEs did not show notable disparity in any subgroup. Further analysis revealed that electrocardiogram QT corrected interval prolonged emerged as the CE with the highest pooled incidence, occurring at a rate of 5.9%.

Although the three HER2-targeted ADCs share the same targeting antibody-trastuzumab, the incidence of cardiotoxicity may also be influenced by factors such as linker stability, intensity of HER2 signal blocking, and pharmacokinetic parameters, etc., which have not been elucidated by studies. The approval of RC48 was based on objective response rates (ORR) observed in single-arm clinical trials, and currently there is a lack of available studies reporting any clinical data regarding cardiac toxicity. A pooled analysis revealed a total rate of 3.37% (95% CI, 2.6-4.3%) for T-DM1-associated cardiotoxicity in advanced HER2-positive breast cancer^66^. Our findings align with this data, indicating a pooled occurrence of 3.6% (95% CI, 2.6-4.6%) for T-DM1-associated cardiotoxicity across different solid tumors, varying dosages, and tumor statuses. The total incidence of T-DXd-associated CEs has not yet been reported. Our research indicated that the total incidence of CEs associated with T-DXd was 7.7% (95% CI, 4.5-10.9%), significantly higher than that of T-DM1. Notably, the incidence of HER2-targeted ADCs is significantly lower than that of trastuzumab^12^, despite the vast majority of patients included in this research were previously exposed to trastuzumab with/without anthracycline. Although we have not yet provided a pharmacological explanation for this difference, the innovative structural design of ADC is believed to be a significant contributing factor.

The combination therapy of T-DM1 or T-DXd in clinical practice is uncommon. Both the American Society of Clinical Oncology (ASCO)^67^ and the National Comprehensive Cancer Network (NCCN)^68^ recommend T-DM1 or T-DXd as a monotherapy. However, the safety and efficacy of T-DM1 combination therapy have been extensively explored in multiple clinical studies included in our paper. The combination drugs include chemotherapeutic agents (such as docetaxel, taxane, nab-paclitaxel, capecitabine, non-pegylated liposomal doxorubicin), HER2 targeting agents (including tucatinib, neratinib, lapatinib, pertuzumab), EGFR inhibitors (such as osimertinib), PI3K inhibitors (such as alpelisib), immune checkpoint inhibitors (atezolizumab and pembrolizumab), as well as cyclin-dependent kinase 4 and 6 (CDK4/6) inhibitor (ribociclib). It is well known that the incidence of CEs was found to be higher when trastuzumab combination therapy, particularly in combination with anthracycline or paclitaxel, at rates of 27% and 13%, respectively^69^. Similarly, our study demonstrated a higher occurrence rate of T-DM1-induced CEs in combination therapy compared to monotherapy (7.8% versus 2.3%; *p*<0.001). There was limited available data on the combination therapy for T-DXd in this paper, and further investigation is necessary to determine whether there will be an increase in the incidence of CEs with combination treatment. The findings of our research served as a reminder for healthcare professionals to exercise caution when considering the combination administration of T-DM1.

The KATHERINE trial[35]established the role of T-DM1 in HER2-positive early breast cancer patients with the residual invasive disease following neoadjuvant therapy. Our research findings indicated a slightly lower incidence of all-grade CEs in early-stage breast cancer compared to advanced/metastatic cases (3.7% versus 5.8%), although this difference did not reach statistical significance (*p*=0.093). Possible factors contributing to this phenomenon include poor performance in advanced/metastatic patients, cumulative administration of anthracyclines, the impact of prior treatments, or potential biases arising from early-stage clinical trials with limited data, etc. Although a higher prevalence of interstitial pneumonia was observed in the high-dose group of T-DXd 70, it has been indicated that varying doses had no impact on treatment-related CEs associated with T-DM1 and T-DXd. Given the small sample size of the phase Ⅰ/Ⅱ trial and the lack of extensive research for tumors other than breast cancer, the comparative results of differences in related subgroups should be interpreted cautiously.

Our findings suggested a low all-grade incidence of CEs associated with HER2-targeted ADC and a lower incidence of severe CE or those leading to treatment discontinuation, which is consistent with current studies^66^, ^70^. Unlike anthracyclines, HER2 targeting agents induce reversible cardiotoxicity characterized by cellular dysfunction and asymptomatic changes in LVEF without inducing cardiomyocyte injury or even death^12^. The most frequently observed CEs associated with HER2-targeted ADC were prolonged corrected QT intervals, with a prevalence rate of 5.9%, which closely aligns with the findings reported by Soares L.R^70^. Prolongation of corrected QT interval is known to be associated with fatal torsade de pointes arrhythmias. However, studies have revealed that both T-DM1^42^ and T-DXd^57^ did not have a clinically significant impact for the prolonged QTc interval remained below the safety threshold of 10 milliseconds. Our research revealed that ejection fraction decrease and congestive cardiac failure were the highest proportion of all-grade and serious CEs, respectively. The instructions of T-DM1 and T-DXd emphasize the significance of regular monitoring of LVEF before and during treatment. Once symptomatic congestive heart failure occurs, permanent discontinuation is recommended.

### Limitations

Our study had several limitations. Firstly, the patient characteristics could not be extracted, including underlying heart disease, baseline LVEF value, cumulative doses of anthracycline, cumulative treatment cycles of previous HER2-targeted agents, etc. Furthermore, the timing, duration, and prognosis of CEs were also unavailable. Consequently, this paper could not discuss the risk factors and characteristics associated with HER2-targeted ADC-induced CEs. Secondly, the limited scope of research on tumors other than breast cancer may introduce a potential bias in subgroup analysis across different tumor types. Last but not least, trials that failed to report CEs were excluded, potentially introducing publication bias and inflating the risk of HER2-targeted ADC-related CEs. On the contrary, the inclusion of all these trials in the study would significantly mitigate the occurrence of CEs, thereby potentially misleading physicians' clinical judgment.

## Conclusions

A meta-analysis of 47 trials involving 10594 patients found that HER2-targeted ADC did not result in a high incidence of CEs at all grades and ≥3/serious grades. Further analysis indicated a significantly higher incidence of all-grade CEs in T-DXd treatment, phase I/II trials and combination therapy. Ejection fraction decreased and cardiac failure (congestive) emerged as the highest proportion of all-grade and ≥3/serious grades CEs, respectively. Our study provide a valuable reference for managing cardiotoxicity. Future real-world data are expected to elucidate further the pathogenesis and population characteristics of HER2-targeted ADC-induced cardiotoxicity.

## Supplementary Material

Supplementary figures and tables.

## Figures and Tables

**Table 1:** General characteristics of included studies in this meta-analysis.

First author	Year	Study name	NCT NO.	Study types	Phase	ADC Drug	Dosage	Cancer	Cancer status	No. of patients	No. of CEs		
											All-grade	≥3/serious grades	Discontinuation
R. Barroso-Sousa^18^	2022	ATEMPT	NCT01853748	RCT	2	T-DM1	3.6 mg/kg q3w	breast	early	383	27	1	3
J. Cortés^19^	2022	DESTINY-Breast03	NCT03529110*	RCT	3	T-DM1	3.6 mg/kg q3w	breast	metastatic	263	1	0	NA
Iwama E^20^	2022	NA	Iwama E, 2022*	single-arm	2	T-DM1	3.6 mg/kg q3w	lung	locally advanced/metastatic	22	1	1	0
Peters, S^21^	2019	NA	NCT02289833	two-arm	2	T-DM1	3.6 mg/kg q3w	lung	locally advanced/metastatic	49	2	0	0
Montemurro, F^22^	2019	KAMILLA	NCT01702571	single-arm	3	T-DM1	3.6 mg/kg q3w	breast	advanced	2002	13	13	NA
Watanabe, J^23^	2017	JO29317	JapicCTI-132395*	single-arm	2	T-DM1	3.6 mg/kg q3w	breast	advanced	232	2	2	2
Krop, I. E^24^	2017	TH3RESA	NCT01419197	RCT	3	T-DM1	3.6 mg/kg q3w	breast	advanced	403	12	1	NA
Thuss-Patience PC^25^	2017	GATSBY	NCT01641939	RCT	2/3	T-DM1	2.4mg/kg qw 3.6 mg/kg q3w	gastric	advanced	293	2	2	NA
Krop, I. E^26^	2015	TDM4874g	NCT01196052*	single-arm	2	T-DM1	3.6 mg/kg q3w	breast	early	148	9	1	1
Hurvitz, S. A^27^	2013	TDM4450g	NCT00679341	RCT	2	T-DM1	3.6 mg/kg q3w	breast	metastatic	69	6	2	NA
Dieras, V^13^	2017	EMILIA	NCT00829166	RCT	3	T-DM1	3.6 mg/kg q3w	breast	advanced	490	5	5	NA
Burris, H. A., 3rd^28^	2011	TDM4258g	NCT00509769*	single-arm	2	T-DM1	3.6 mg/kg q3w	breast	metastatic	112	2	0	0
Beeram, M^29^	2012	TDM3569g	NCT00932373	single-arm	1	T-DM1	0.3-4.8 mg/kg q3w 1.2 -2.9mg/kg qw	breast	advanced	52	1	NA	NA
Krop, I. E^30^	2012	TDM4374g	NCT00679211	single-arm	2	T-DM1	3.6 mg/kg q3w	breast	metastatic	110	1	1	0
de Vries, E. G. E^31^	2023	KAMELEON	NCT02999672	single-arm	2	T-DM1	2.4 mg/kg qw 3.6 mg/kg q3w	UC/PC/CAA	advanced	20	1	0	0
Yardley, D. A^32^	2015	TDM4884g	NCT01120561*	single-arm	NA	T-DM1	3.6 mg/kg q3w	breast	metastatic	215	14	4	4
Kashiwaba, M^33^	2016	JO22997 study	JAPIC CTI-101277*	single-arm	2	T-DM1	3.6 mg/kg q3w	breast	locally advanced/recurrent /metastatic	73	2	0	1
Yamamoto, H^34^	2015	NA	Yamamoto H,2015*	single-arm	1	T-DM1	3.6 mg/kg q3w	breast	metastatic	10	2	0	0
Huang, C. S^35^	2021	KATHERINE	NCT01772472*	RCT	3	T-DM1	3.6 mg/kg q3w	breast	residual invasive disease after neoadjuvant	740	23	4	NA
Cortes, J^36^	2020	TRAXHER2	NCT01702558	RCT	1/2	T-DM1 + /-capecitabine	2.4/ mg/kg qw 3.6 mg/kg q3w	gastric/breast	locally advanced/metastatic	178	10	1	NA
Martin, M^37^	2016	BP22572	NCT00934856	non-RCT	1/2	T-DM1 + docetaxel+/-P	2.4mg/kg qw 3.6 mg/kg q3w	breast	locally advanced/metastatic	98	3	0	0
Perez, E. A^38^	2019	MARIANNE	NCT01120184	RCT	3	T-DM1 +/-P	3.6 mg/kg q3w	breast	advanced	727	28	5	NA
Abraham, J^39^	2019	NA	NCT02236000	single-arm	1/2	T-DM1+ neratinib	3.6 mg/kg q3w	breast	metastatic	76	4	0	NA
Jebbink, M^40^	2023	TRAEMOS	NCT03784599*	single-arm	1/2	T-DM1+ osimertinib	3.6 mg/kg q3w	lung	metastatic	27	1	0	1
Emens, L. A^41^	2020	KATE2	NCT02924883*	RCT	2	T-DM1+/-atezolizumab	3.6 mg/kg q3w	breast	metastatic	200	2	2	NA
Gupta, M^42^	2013	TDM4688g	NCT00943670	single-arm	2	T-DM1+/-P	3.6 mg/kg q3w	breast	metastatic	71	8	1	1
Jain, S^43^	2018	NA	NCT02038010	single-arm	1	T-DM1+alpelisib	3.6 mg/kg q3w	breast	metastatic	17	15	0	NA
Patel, T. A^44^	2019	TEAL	NCT02073487	RCT	2	T-DM1+lapatinib+nab-paclitaxel	3.0 mg/kg q3w	breast	stage II to III	14	1	0	NA
Lopez-Miranda, E^45^	2020	THELMA	NCT02562378	single-arm	1	T-DM1+non-pegylated liposomal doxorubicin	3.6 mg/kg q3w	breast	metastatic	15	13	0	0
Krop, I. E^46^	2022	KAITLIN	NCT01966471	RCT	3	TDM-1+P	3.6 mg/kg q3w	breast	early	912	36	7	NA
Hurvitz, S. A^47^	2019	KRISTINE	NCT02131064	RCT	3	T-DM1+P	3.6 mg/kg q3w	breast	stage II to III	223	1	1	NA
Miller, K. D^48^	2014	NA	NCT00875979*	single-arm	1/2	T-DM1+P	3.6 mg/kg q3w	breast	locally advanced/metastatic	64	2	2	1
Filho, O. M^49^	2021	NA	Filho OM, 2021*	single-arm	2	T-DM1+P	3.6 mg/kg q3w	breast	early	163	5	0	NA
Krop, I. E^50^	2016	TDM4652g	NCT00951665	single-arm	1/2	T-DM1+paclitaxel ±P	1.2-2.4 mg/kg qw 2.0-3.6mg/kg q3w	breast	metastatic	104	2	2	0
Waks, A. G^51^	2022	NA	NCT03032107*	single-arm	1	T-DM1+pembrolizumab	3.6 mg/kg q3w	breast	metastatic	20	1	0	NA
Spring, L. M^52^	2021	NA	NCT02657343*	single-arm	1	T-DM1+ribociclib	3.6 mg/kg q3w	breast	advanced/metastatic	12	5	0	NA
Borges, V. F^53^	2018	NA	NCT01983501*	single-arm	1	T-DM1+tucatinib	3.6 mg/kg q3w	breast	advanced	57	2	0	2
Cortes, J^19^	2022	DESTINY-Breast03	NCT03529110*	RCT	3	T-DXd	5.4 mg/kg q3w	breast	metastatic	261	7	0	NA
Modi, S^54^	2020	DESTINY-Breast01	NCT03248492	single-arm	2	T-DXd	5.4 mg/kg q3w	breast	metastatic	184	14	1	0
Andre, F^55^	2023	DESTINY-Breast02	NCT03523585*	RCT	3	T-DXd	5.4 mg/kg q3w	breast	metastatic	406	18	2	2
Modi, S^14^	2022	DESTINY-Breast04	NCT03734029*	RCT	3	T-DXd	5.4 mg/kg q3w	breast	metastatic	371	63	7	NA
Bartsch, R^56^	2022	TUXEDO-1 trial	NCT04752059*	single-arm	2	T-DXd	5.4 mg/kg q3w	breast	metastases	15	1	1	1
Shimomura, A^57^	2023	DS8201-A-J102	NCT03366428	single-arm	1	T-DXd	6.4 mg/kg q3w	breast	metastatic	51	7	0	1
Chang D.Y^58^	2019	NA	NCT03368196	single-arm	1	T-DXd	6.4 mg/kg q3w	breast/gastric	advanced	12	1	0	0
Tamura K^59^, Modi, S^60^, Shitara, K^61^, Tsurutani, J^62^, Doi, T^63^	2019	J101	NCT02564900	single-arm	1	T-DXd	5·4/ 6·4 mg/kg q3w 0.8-8.0mg/kg q3w	breast/gastric/other solid tumors	advanced/metastatic	299	42	0	0
Yamaguchi, K^64^	2023	DESTINY-Gastric01	NCT03329690*	RCT	2	T-DXd	6.4 mg/kg q3w	gastric	advanced	169	4	3	NA
Li, B. T^7^	2022	DESTINY-Lung01	NCT03505710	single-arm	2	T-DXd	5.4/6.4 mg/kg q3w	lung	relapsed/refractory	181	10	2	NA
Takahashi, S^65^	2021	NA	NCT03383692	single-arm	1	T-DXd +Ritonavir/Itraconazole	5.4 mg/kg q3w	solid tumor	unresectable/metastatic	40	2	0	NA

Abbreviations: CE: cardiac event, NA: not available;*data extracted from published articles or abstracts.

**Table 2:** Subgroup analysis for the incidence of all-grade HER2-targeted ADC-related cardiac events.

Subgroup variables	No. of trials	No. of patients	Pooled ES (95% CI)	Measure of heterogeneity	
				*p*	I^2^
**ADC Drug**				0.017*	/
T-DM1	37	8664	0.036 (0.026-0.046)	<0.001	90.003%
T-DXd	11	1945	0.077 (0.045-0.109)	<0.001	86.798%
**Phase of trials**				0.002*	/
Ⅰ/Ⅱ	35	3303	0.069 (0.049,0.089)	<0.001	89.244%
III	10	6798	0.032 (0.019,0.045)	<0.001	93.437%
**Combination therapy**				0.013*	/
No	32	7888	0.039(0.030,0.049)	<0.001	86.641%
Yes	19	2460	0.076 (0.049,0.104)	<0.001	93.201%
**Cancer type**				0.787*	/
Breast cancer	38	9534	0.055 (0.037,0.076)	<0.001	91.727%
Lung cancer	4	279	0.045(0.021,0.075)	0.994	0.000%
Gastric cancer	3	462	0.030(0.000,0.091)	/	/
Other solid tumor	2	60	0.048(0.003,0.125)	/	/
**Cancer status**				0.093*	/
Early-stage breast cancer	7	2583	0.037(0.018,0.056)	<0.001	86.008%
advanced/metastatic stage breast cancer	32	6963	0.058(0.043,0.073)	<0.001	92.527%
**Dosage for T-DM1**				0.933*	/
3.6 mg/kg q3w	34	8178	0.043(0.027,0.062)	<0.001	89.187%
Less than 3.6 mg/kg q3w	3	253	0.038(0.000,0.191)	/	/
**Dosage for T-DXd**				0.933*	/
5.4 mg/kg q3w	7	1386	0.072(0.028,0.130)	<0.001	89.935%
6.4 mg/kg q3w	4	232	0.071(0.014,0.159)	0.011	73.305%

Abbreviations: ES: Effect Size, CI: confidence interval, *test for subgroup differences.Statistically significant values are marked in boldface.

**Table 3:** Subgroup analysis for the incidence of ≥3/serious grades HER2-targeted ADC-related cardiac events.

Subgroup variables	No. of trials	No. of patients	Pooled ES (95% CI)	Measure of heterogeneity	
				*p*	I^2^
**ADC Drug**				0.307*	/
T-DM1	20	6920	0.006 (0.004,0.008 )	0.621	0.000%
T-DXd	6	1192	0.010 (0.003,0.017)	0.270	21.735%
**Phase of trials**				0.969*	/
Ⅰ/Ⅱ	35	3303	0.006 (0.002,0.010)	0.502	0.000%
III	10	6798	0.006 (0.004,0.008)	0.394	4.910%
**Combination therapy**				0.239*	/
No	19	6367	0.005 (0.003,0.009)	0.127	27.834%
Yes	8	1745	0.004 (0.000,0.015)	0.046	51.013%
**Cancer type**				0.197*	/
Breast cancer	21	7575	0.005 (0.002,0.008)	0.081	31.863%
Lung cancer	2	113	0.021(0.000,0.062)	/	/
Gastric cancer	2	418	0.010(0.002,0.023)	/	/
**Cancer status**				0.379*	/
Early-stage breast	4	2583	0.005(0.002,0.008)	0.620	0.000%
advanced/metastatic stage breast	19	6963	0.007(0.005,0.009)	0.450	0.112%

Abbreviations: ES: Effect Size, CI: confidence interval. *test for subgroup differences.

**Table 4:** Types of cardiac events associated with HER2-targeted ADC

Types	No. of trials	No. of patients	No. of All- grade CEs	No. of ≥3/serious grades CEs	Pooled analysis of all-grade CEs		
					ES (95% CI)	*p*	I^2^
Ejection fraction decrease	17	4443	173	11	0.033(0.022,0.044)	<0.001	75.991%
Electrocardiogram QT corrected interval prolonged	11	639	50	0	0.059(0.033,0.085)	0.015	54.414%
Palpitations	10	3296	40	2	0.027(0.010,0.044)	<0.001	75.623%
Left ventricular systolic dysfunction	6	1207	23	4	0.014(0.001,0.028)	0.003	72.114%
Tachycardia	7	1046	22	2	0.025(0.005,0.044)	0.005	67.708%
Sinus tachycardia	7	1933	19	2	0.016(0.000,0.052)	<0.001	88.174%
Cardiac failure(congestive)	14	4867	18	14	0.001(0.000,0.002)	0.432	1.546%
Atrial fibrillation	6	1407	6	6	0.003(0.000,0.006)	0.884	0.000%
Pericardial effusion	6	2625	8	5	0.002(0.000,0.016)	0.003	71.745%
Troponin increased	2	106	7	1	/	/	/
Brain natriuretic peptide increased	1	15	6	0	/	/	/
Acute coronary syndrome	2	2308	5	5	/	/	/
Cardiomyopathy	3	1527	3	3	/	/	/
Supraventricular tachycardia	3	2751	4	3	/	/	/
Cardic arrest	2	2022	3	2	/	/	/
Sinus bradycardia	3	1312	3	1	/	/	/
Atrial thrombosis	2	223	2	2	/	/	/
Ventricular tachycardia	2	749	2	1	/	/	/
Supraventricular extrasystoles	2	217	2	1	/	/	/
Nonspecific T Wave Abnormality on ECG	2	58	2	1	/	/	/
Myocardial infarction	2	20	2	1	/	/	/
Other*	13	4485	40	6	/	/	/

*include ventricular fibrillation, extrasystoles, angina pectoris , ventricular dysfunction, cardiac tamponade,pericarditis1, Chest pain-cardiac, cardio-respiratory arrest , pulmonary edema , right bundle block, tricuspid valve incompetence, mitral valve incompetence etc. Abbreviations: CE: cardiac event, ES: Effect Size, CI: confidence interval
