# Supplementary material for: Incidence of HER2-targeted antibody-drug conjugates-related cardiac events: a meta-analysis
Source: J Cancer. 2024 Jan 1;15(1):90–102. doi: 10.7150/jca.90090 (PMC10751674; doi:10.7150/jca.90090)
Supplement: Supplementary file 1 — Supplementary figures and tables. [file jcav15p0090s1.pdf]

**SUPPLEMENTARY MATERIAL**

Table of Contents

Figure S1. Risk of bias assessment for RCT according to RoB tool

Figure S2. The incidence of all-grade cardiac toxicity between phase I/II trials and phase III trials in different drug subgroups.

Figure S3. The incidence of cardiac toxicity between T-DM1 and T-DXd in monotherapy treatment.

Figure S4. The incidence of cardiac toxicity of T-DM1 in monotherapy and combination therapy.

Figure S5. Funnel plots of the incidence of cardiotoxicity for all grades (A) and ≥3/serious grades(B).

Table S1. Search strategy via PubMed up to May 2023.

Table S2. A list of MINORS scale scores for non-randomized controlled trials.

A

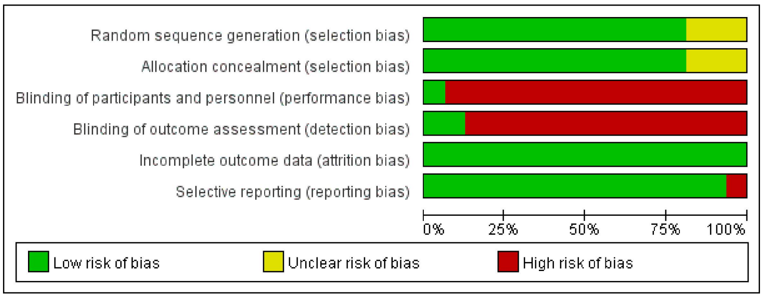


B

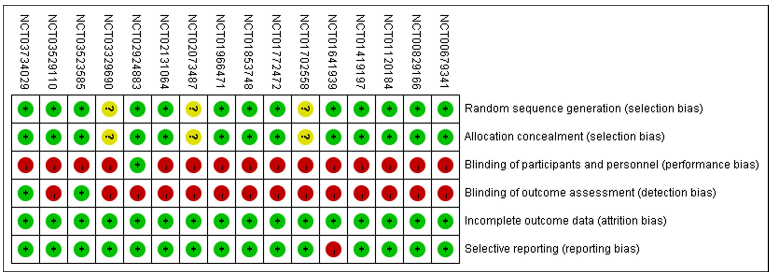


Figure S1. Risk of bias assessment for RCT according to RoB tool. (A) Risk of bias graph; (B) Risk of bias summary.

A

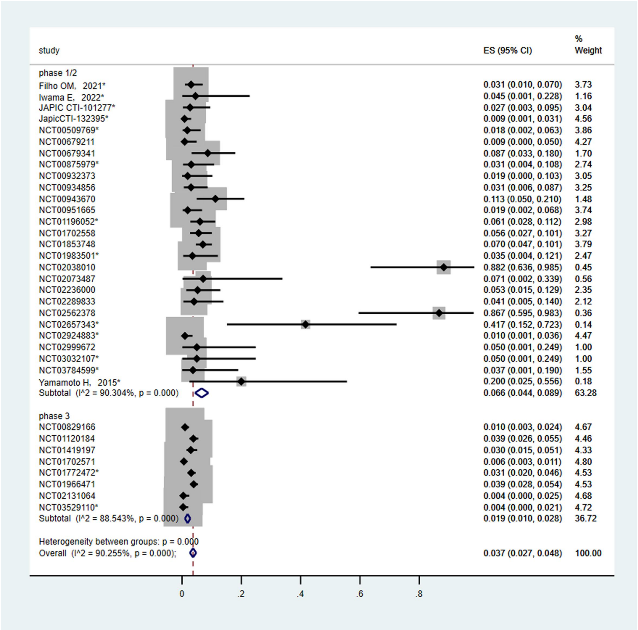

B

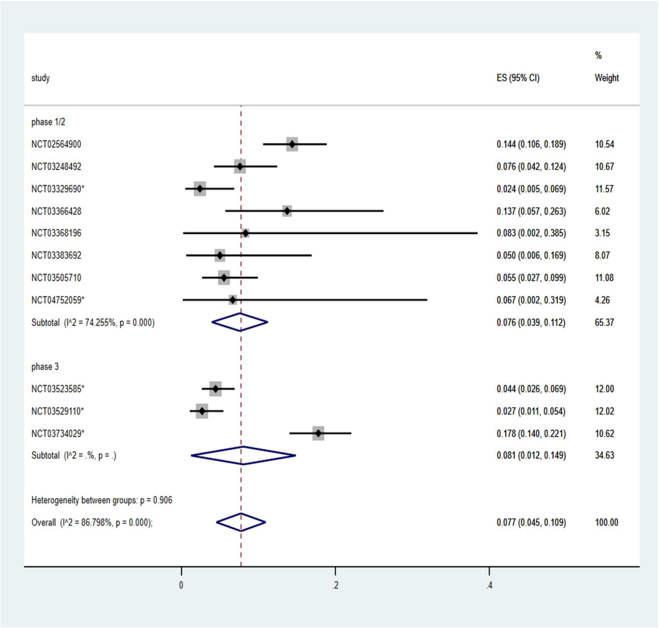

Figure S2. The incidence of all grade cardiac toxicity between phase I/II trials and phase III trials in different drug subgroups. (A) The incidence of all-grade cardiac toxicity between phase I/II trials and phase III trials in T-DM1 treatment. (B) The incidence of all-grade cardiac toxicity between phase I/II trials and phase III trials in T-DXd treatment.

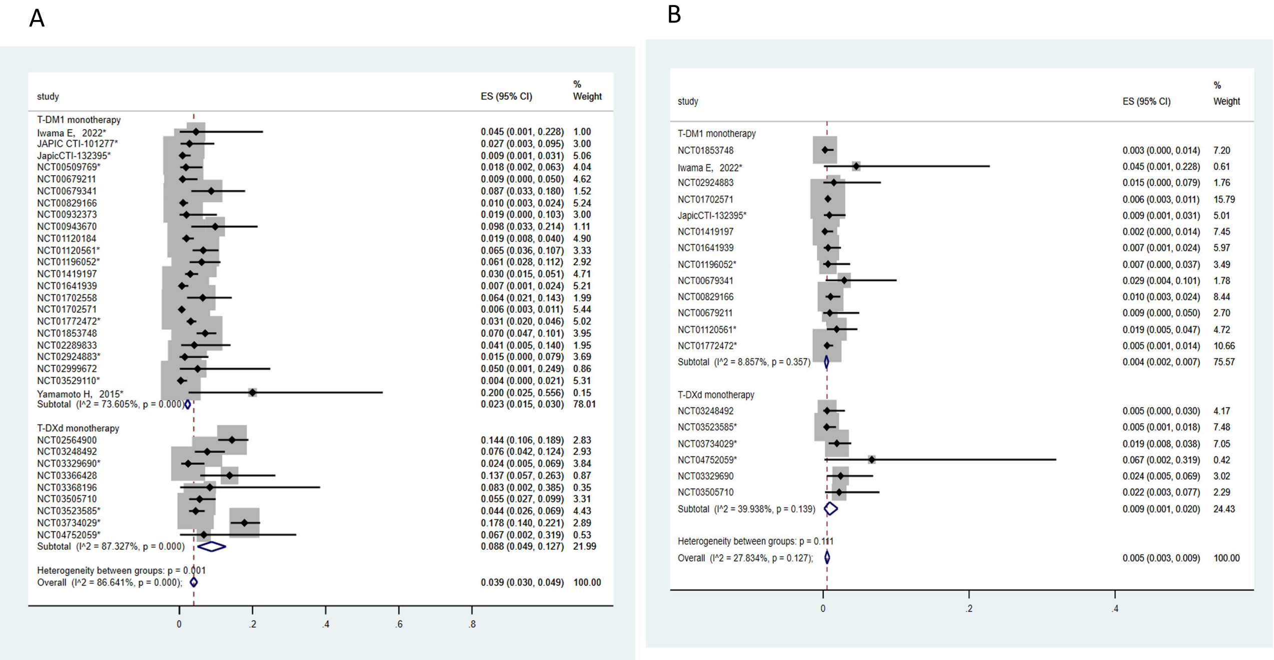


Figure S3. The incidence of cardiac toxicity between T-DM1 and T-DXd in monotherapy treatment. (A)The incidence of all-grade cardiac toxicity between T-DM1 and T-DXd in monotherapy treatment. (B)The incidence of ⩾3/serious grades cardiac toxicity between T-DM1 and T-DXd in monotherapy treatment.

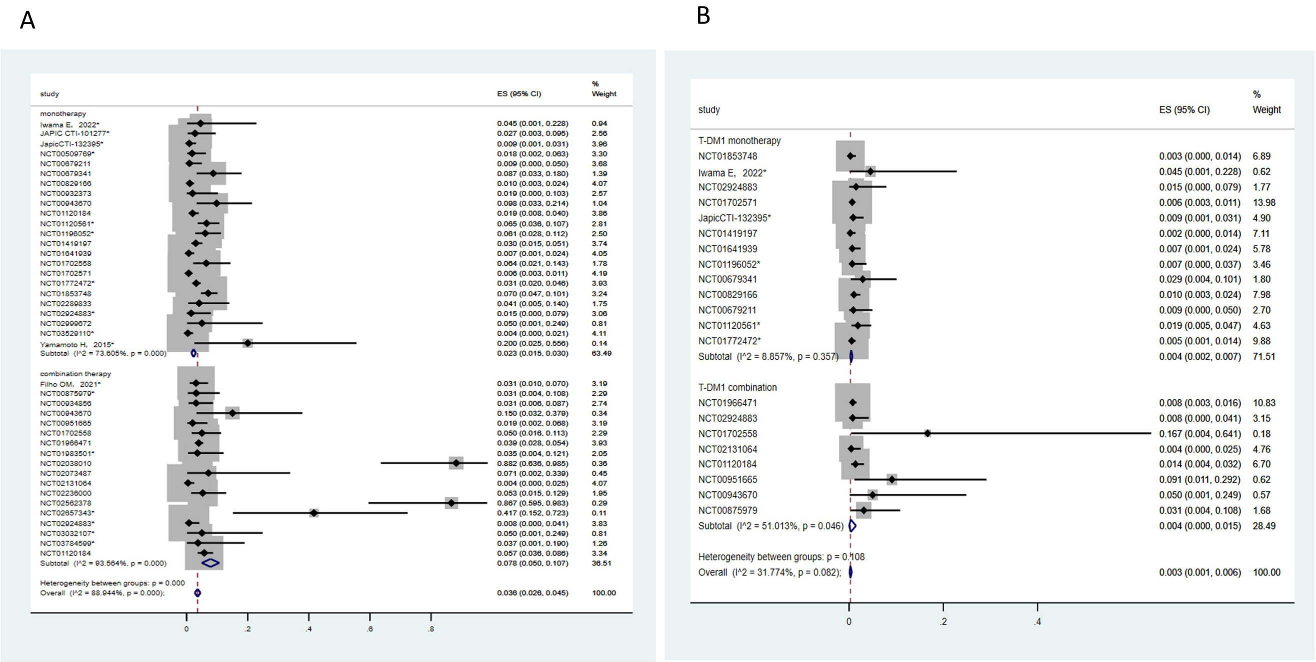


Figure S4. The incidence of cardiac toxicity of T-DM1 in monotherapy and combination therapy. (A) The incidence of all-grade cardiac toxicity induced by T-DM1 in monotherapy and combination therapy. (B) The incidence of cardiac toxicity with ⩾3/serious grades induced by T-DM1 in monotherapy and combination therapy.

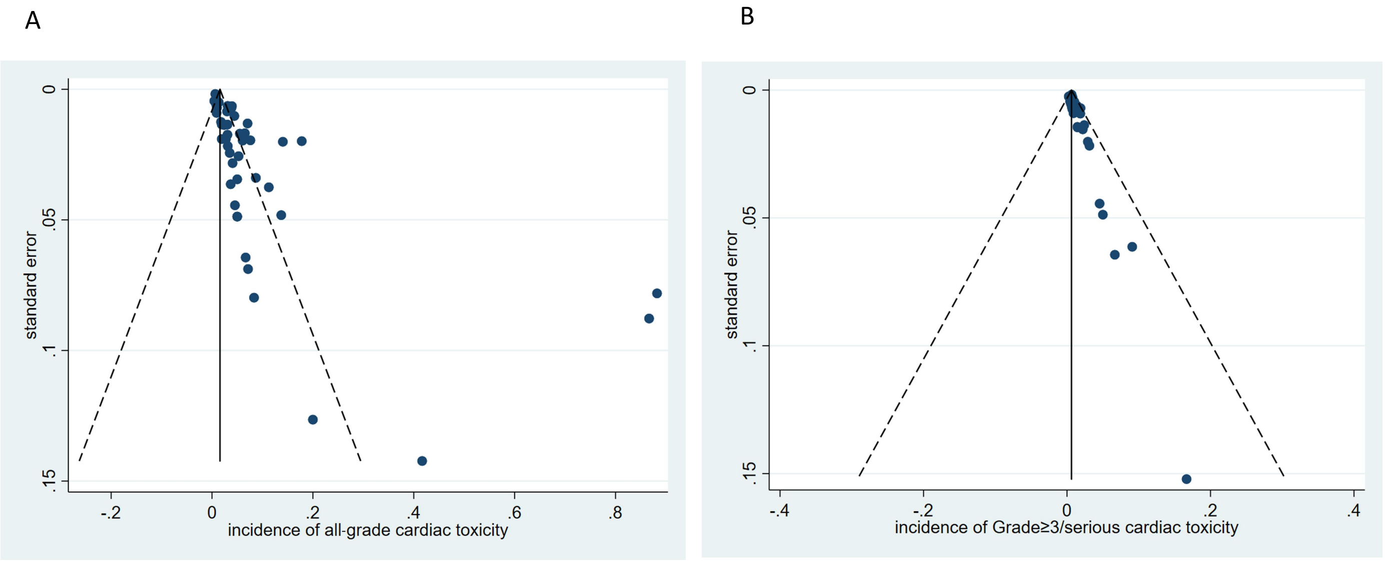


Figure S5. Funnel plots of the incidence of cardiotoxicity for all grades (A) and ⩾3/serious grades (B).

Table S1. Search strategy via PubMed up to May 2023

| number | Search strategy | Search results(items) |
|---|---|---|
| #1 | (disitamab vedotin[Title/Abstract]) OR(rc 48[Title/Abstract]) OR (aidixi[Title/Abstract]) | 18 |
| #2 | ("Ado-Trastuzumab Emtansine"[Mesh]) OR ((((((((((Ado Trastuzumab Emtansine[Title/Abstract]) OR (Trastuzumab Emtansine[Title/Abstract])) OR (Kadcyla[Title/Abstract])) OR (huN901-DM1[Title/Abstract])) OR (huN901DM1[Title/Abstract])) OR (Trastuzumab-DM1 Conjugate[Title/Abstract])) OR (Trastuzumab DM1 Conjugate[Title/Abstract])) OR (Trastuzumab-DM1[Title/Abstract])) OR (Trastuzumab DM1[Title/Abstract])) | 1071 |
| #3 | (trastuzumab deruxtecan [Supplementary Concept]) OR ((((((((ds 8201[Title/Abstract])) OR (fam-trastuzumab deruxtecan-nxki[Title/Abstract])) OR (ds 8201a[Title/Abstract])) OR (ds8201[Title/Abstract])) OR (ds8201a[Title/Abstract])) OR (enhertu[Title/Abstract])) OR (fam trastuzumab deruxtecan nxki[Title/Abstract])) OR (trastuzumab deruxtecan[Title/Abstract])) | 361 |
| #4 | #1 OR #2 OR #3 | 1354 |

Table S2. A list of MINORS scale scores for non-randomized controlled trials .

| study | A clearly stated aim | Inclusion of consecutive patients | Prospective collection of data | Endpoints appropriate to the aim of the study | Unbiased evaluation of endpoints | Follow-up period appropriate to the aim of the study | Loss to follow up less than 5% | Prospective calculation of the study size | An adequate control group | Contem porary groups | Baseline equivalence of groups | Adequate statistical analyses | scores† |
|---|---|---|---|---|---|---|---|---|---|---|---|---|---|
| Iwama E，2022 | 2 | 2 | 2 | 1 | 0 | 2 | 2 | 2 | / | / | / | / | 13 |
| NCT02289833 | 2 | 2 | 2 | 1 | 0 | 2 | 2 | 2 | / | / | / | / | 13 |
| NCT01702571 | 2 | 2 | 2 | 1 | 0 | 2 | 0 | 0 | / | / | / | / | 9 |
| JapicCTI-132395 | 2 | 2 | 2 | 1 | 0 | 1 | 0 | 0 | / | / | / | / | 8 |
| NCT01196052 | 2 | 0 | 2 | 1 | 0 | 2 | 2 | 2 | / | / | / | / | 11 |
| NCT00509769 | 2 | 2 | 2 | 2 | 0 | 2 | 2 | 0 | / | / | / | / | 12 |
| NCT00932373 | 2 | 1 | 2 | 1 | 0 | 1 | 2 | 0 | / | / | / | / | 9 |
| NCT00679211 | 2 | 2 | 2 | 2 | 0 | 2 | 2 | 2 | / | / | / | / | 14 |
| NCT02999672 | 1 | 2 | 2 | 1 | 0 | 2 | 2 | 0 | / | / | / | / | 10 |
| NCT01120561 | 2 | 2 | 2 | 2 | 0 | 1 | 2 | 0 | / | / | / | / | 11 |
| JAPIC CTI-10127 | 2 | 1 | 2 | 1 | 0 | 1 | 2 | 2 | / | / | / | / | 11 |
| Yamamoto H,2015 | 2 | 2 | 2 | 1 | 0 | 1 | 2 | 0 | / | / | / | / | 10 |
| NCT00934856 | 2 | 2 | 2 | 2 | 0 | 0 | 2 | 0 | / | / | / | / | 10 |
| NCT02236000 | 2 | 2 | 2 | 1 | 0 | 1 | 2 | 0 | / | / | / | / | 10 |
| NCT03784599 | 2 | 2 | 2 | 1 | 0 | 2 | 2 | 2 | / | / | / | / | 13 |
| NCT00943670 | 2 | 2 | 2 | 2 | 0 | 1 | 2 | 0 | / | / | / | / | 11 |
| NCT02038010 | 2 | 2 | 2 | 1 | 0 | 2 | 0 | 2 | / | / | / | / | 11 |
| NCT02562378 | 2 | 2 | 2 | 2 | 0 | 2 | 2 | 0 | / | / | / | / | 12 |
| NCT00875979 | 2 | 2 | 2 | 2 | 0 | 0 | 2 | 2 | / | / | / | / | 12 |
| Filho OM，2021 | 2 | 2 | 2 | 1 | 0 | 0 | 2 | 2 | / | / | / | / | 11 |
| NCT00951665 | 1 | 2 | 2 | 1 | 0 | 0 | 2 | 0 | / | / | / | / | 8 |
| NCT03032107 | 2 | 2 | 2 | 2 | 0 | 2 | 2 | 0 | / | / | / | / | 12 |
| NCT02657343 | 2 | 2 | 1 | 1 | 0 | 1 | 2 | 0 | / | / | / | / | 9 |
| NCT01983501 | 2 | 2 | 2 | 2 | 0 | 1 | 2 | 0 | / | / | / | / | 11 |
| NCT03248492 | 2 | 2 | 2 | 2 | 0 | 1 | 2 | 2 | / | / | / | / | 11 |
| NCT04752059 | 2 | 2 | 2 | 2 | 0 | 2 | 2 | 2 | / | / | / | / | 14 |
| NCT03366428 | 2 | 2 | 2 | 2 | 0 | 2 | 2 | 2 | / | / | / | / | 14 |
| NCT03368196 | 2 | 2 | 2 | 2 | 0 | 0 | 2 | 0 | / | / | / | / | 10 |
| NCT02564900 | 2 | 2 | 2 | 2 | 0 | 1 | 2 | 2 | / | / | / | / | 13 |
| NCT03505710 | 2 | 2 | 2 | 2 | 0 | 1 | 2 | 2 | 2 | 2 | 2 | 2 | 21 |
| NCT03383692 | 2 | 2 | 2 | 2 | 0 | 1 | 1 | 2 | / | / | / | / | 12 |

MINORS：Methodological index for non-randomized studies；†The items are scored 0 (not reported), 1 (reported but inadequate) or 2 (reported and adequate). Item 1-8 for non-comparative studies,item 1-12 for comparative studies. The global ideal score being 16 for non-comparative studies and 24 for comparative studies.
